# Development of an ecological framework for building successful collaboration between Primary Care and Public Health

**DOI:** 10.1186/1472-6963-14-S2-P133

**Published:** 2014-07-07

**Authors:** Ruta Valaitis, Marjorie MacDonald, Sabrina Wong, Ruth Martin-Misener, Linda O’Mara, Donna Meagher-Stewart

**Affiliations:** 1 McMaster University, Hamilton, ON, Canada; 2 University of Victoria, Victoria, BC, Canada; 3 University of British Columbia, Vancouver, BC, Canada; 4 Dalhousie University, Halifax, NS, Canada

## Background

Health systems worldwide are interested in determining the best ways for primary care (PC) and public health (PH) to collaborate to improve population and system outcomes. Since examples of successful collaborations between PC and PH exist, research is needed to document what has worked and lessons learned. This presentation will describe the development of the Ecological Framework for Building Successful Collaboration Between Primary Care and Public Health. The Framework is the culmination of a four and a half year program of research that aimed to: explore structures and processes required to build successful collaborations between PC and PH; understand the nature of existing collaborations in Canada; and, examine roles that nurses and other providers played in collaborations.

## Materials and methods

Five consecutive research projects informed the Framework’s development which included: 1) an international scoping literature review; 2) environmental scans in three Canadian provinces; 3) a descriptive interpretive study with key informants from across Canada and PC and PH sectors; 4) Q-sort methodology to identify common viewpoints of stakeholders, and; 5) a multiple case study involving 10 cases in three provinces.

Study settings included British Colombia, Ontario and Nova Scotia and involved direct service providers, policymakers, administrators and managers from PC and PH sectors.

## Results

The Framework represents the nature of PC and PH collaboration and factors that can influence the development and maintenance of successful collaborations. The nature of collaboration, which is found at the core of the framework, is the structure and context around which the collaboration is formed. Factors influencing collaboration exist at the intrapersonal, interpersonal, organizational and systemic levels.

## Conclusions

The Framework informs the development and maintenance of successful collaborations between PC and PH and evaluation of collaborations relevant to policy makers, managers and front line providers and may have application to collaborations beyond PC and PH sectors.

